# Type 2 Diabetes and Oxidative Stress and Inflammation: Pathophysiological Mechanisms and Possible Therapeutic Options

**DOI:** 10.3390/antiox11050953

**Published:** 2022-05-12

**Authors:** Cristina Vassalle, Melania Gaggini

**Affiliations:** 1 Fondazione CNR-Regione Toscana G Monasterio, 56124 Pisa, Italy; 2 Istituto di Fisiologia Clinica, Consiglio Nazionale delle Ricerche-CNR, 56124 Pisa, Italy; mgaggini@ifc.cnr.it

Type 2 diabetes (T2D) is a public health burden associated with high healthcare and societal costs and elevated morbidity and mortality [1]. The number of T2D patients has quadrupled in the past three decades, and this disease represents the ninth major cause of death [2].

In addition to a correct lifestyle, including maintaining a healthy body weight, a proper diet, physical activity, not smoking/smoking cessation, and traditional pharmacological interventions (e.g., insulin, metformin), newer diabetes mellitus drug classes, i.e., sodium-glucose transport protein 2 (SGLT2) inhibitors and Glucagon-Like Peptide-1 Receptor Agonists (GLP-1Ras) are recommended in recent guidelines as the add-on therapy to metformin to improve glycaemic control [3]. These pharmacological agents are particularly advantageous for patients with cardiovascular disease, as these drugs also retain cardiovascular benefits that appear independent of glycaemic control and of baseline metformin use [4,5]. There is strong evidence of interrelationships between oxidative stress and inflammation and molecular and cellular events related to T2D onset, progression, and complications [6,7]. Interestingly, different agents may target inflammatory and pro-oxidant pathways related to T2D, providing additional potential therapeutic benefits for the treatment of T2D [8]. Five research studies addressed different aspects of this important topic in this Special Issue.

Canet et al. investigated the effects of empagliflozin (10 mg/day for 24 weeks), a novel potent and selective drug belonging to SGLT-2, on anthropometric and endocrine parameters, leukocyte–endothelium interactions, adhesion molecules, reactive oxygen species (ROS) production, and NFkB-p65 transcription factor expression in T2D patients. The results evidenced the anti-inflammatory and antioxidant properties of empagliflozin (decrease in intercellular adhesion molecule-1, mitochondrial reactive oxygen species (ROS) levels, and interleukine-6 and NFkB-p65 expression, as well as an increase in superoxide dismutase together with waist circumference, body weight, BMI, glycated haemoglobin, and glucose reduction) [9]. Moreover, a reduction in leukocyte–endothelium interactions, rolling flux, and adhesion at 24 weeks was also observed in in vitro experiments (HUVEC cells). The results obtained highlight the benefit of this drug against the atherosclerotic and inflammatory processes, with a possible reduction in cardiovascular events in T2D patients.

In the study of Wu et al., the pharmacological prevention of cataracts, a common T2D complication, was investigated in an experimental model of fructose-induced diabetic rats by the administration of 3H-1,2-Dithiole-3-Thione (D3T 10 mg/kg/day, 8 weeks) [10]. This treatment elicited a protection of lens epithelial cells against fructose-induced epithelial–mesenchymal transition through the activation of AMPK to reduce reduced superoxide generation and counteract aldose reductase (AKR1B1)-induced oxidative stress. These results entice further D3T evaluation in T2D clinical settings as a potential candidate for pharmacological prevention of cataract.Kim et al. evaluated the role of substance-P (SP), an endogenous peptide involved in cell proliferation and migration by activating survival-related signalling pathways, in cardiac microvascular endothelial cells (CMECs) subjected to high glucose-induced oxidative stress [11]. They found that SP treatment (100 nM) was able to restore the high glucose-mediated adverse effects (reduced viability, oxidative stress, inactivation of PI3/Akt signalling, and loss of vasculature-forming ability). This in vitro study opens to further evaluation of therapeutic potential of SP to treat diabetic complications through its antioxidant properties.

Chen HJ et al. investigated the properties of rice husk silica liquid (RHSL), derived from rice husk, in RIN-m5F pancreatic β cells after streptozotocin (STZ)-stimulation [12]. In particular, RHSL administration (different dosage 100–400 fold dilution) is able to reverse cell viability, insulin secretion, ROS production, change in mitochondria depolarization, and also reduce STZ-induced apoptosis by inducing autophagy in RIN-m5F cells. This results suggest the beneficial effect of RHSL on pancreatic β cells that, and in view of its antioxidant and other properties, merits further evaluation in studies designed to improve T2D and its complications.

The manuscript of Chen LJ et al. evidenced the antioxidant and anti-inflammatory effects of rutin, a flavonoid contained in many plants, on wound healing in streptozotocin-induced hyperglycaemic rats [13]. The beneficial effects of this substance (100 mg/kg body weight intraperitoneal) are evidenced by the production of antioxidant enzymes induced by nuclear factor erythroid 2-related factor 2, inhibition of the expression of matrix metalloproteinases regulated by NF-κB, reduced expression of vascular endothelial growth factor, and promotion of the expression of neurogenic-related protein. Moreover, intraperitoneal injection of rutin significantly improved diabetes-induced body weight loss and metabolic dysfunctions (liver enzymes, lipid profile, and glucose and insulin levels) of hyperglycaemic rats. Thus, the results of these experimental studies support the potential of rutin to prevent or treat pathologies associated with T2D.

The Special Issue is completed by two reviews on the role of oxidative stress and inflammation on two aspects generally little to not at all considered in the clinical practice: differences between female and male patients, still undervalued in disease management, and air pollution exposure, also neglected because, although it may impact on individual risk, it is difficult to quantify in the single patient as sufficiently accurate individual exposure is arduous to be estimated.

Although it is known that gender is a critical determinant for disease prevention onset, progression and outcomes, as well as response to treatments, gender bias in healthcare is common. This is in part due to the pre-existing male imbalance in scientific literature that consists in fewer females included in clinical studies. Consequently, results obtained in males are applied to female patients, where they cannot adequately fit. However, the finality of the “sex/gender medicine” perspective is to address not only the women-man dichotomy but to also understand the differences existing between the two sexes/genders and consider the sex/gender concept as an important modifier that should be considered in medicine and healthcare decision making. This is true also for T2D, where there is an evident lack of knowledge on gender effects in determining T2D and its development. The review of Contreras-Zentella et al. deals with the relationship between obesity, inflammation, insulin resistance, T2D, and inflammation and oxidative stress, evidencing the gender-related differences (e.g., major vulnerability of men to metabolic syndrome compared to premenopausal women, reduced when considering postmenopausal women; higher insulin sensitivity in women; fatty liver index, liver enzymes, triglycerides, waist circumference, and body mass index as better indicators of metabolic syndrome in women than in men; inflammatory and cytokines higher in women; nitrogen metabolism by erythrocytes and vascular nitric oxide activity more increased and decreased, respectively, in diabetic men) [14]. A better knowledge of these differences is expected to increase gender equity in disease management and improve quality in clinical practice, helping to develop more efficacious preventive, diagnostic, and therapeutic tools specifically targeted to female and male patients.

Among T2D determinants, many data have documented the adverse effects of environmental factors (e.g., air pollutants) through multiple exposure-induced mechanisms (e.g., systemic inflammation and oxidative stress, hypercoagulability, and endothelial and immune responses). Gorini et al. discuss the role of air pollution in oxidative stress adverse effects on glycaemic metabolism homeostasis, with a particular focus on its impact on health. In this context, the improvement of new advanced tools (e.g., omic techniques and the study of epigenetic changes) may offer a substantial contribution, providing a road map for future research. The review gives a comprehensive assessment of the molecular, clinical, environmental, and epidemiological factors, underlying the importance of air quality and providing a rationale to implement measures aimed to decrease air pollution-related T2D risk [15].

Overall, articles included in the present Special Issue cover valuable insights for better understanding T2D pathophysiological mechanisms, giving future perspective of possible therapeutic options in view of the relationship between T2D and oxidative stress and inflammation. Editors strongly wish that it could be an opportunity for readers to dwell on challenges and potential future directions of these developing fields in T2D management.
